# Cholesterol stimulates the cellular uptake of L-carnitine by the carnitine/organic cation transporter novel 2 (OCTN2)

**DOI:** 10.1074/jbc.RA120.015175

**Published:** 2021-01-05

**Authors:** Lu Zhang, Ting Gui, Lara Console, Mariafrancesca Scalise, Cesare Indiveri, Stephanie Hausler, Gerd A. Kullak-Ublick, Zhibo Gai, Michele Visentin

**Affiliations:** 1 College of Traditional Chinese Medicine, Innovation Research Institute of Traditional Chinese Medicine, Shandong University of Traditional Chinese Medicine, Jinan, China; 2 Department of Clinical Pharmacology and Toxicology, University Hospital Zurich, University of Zurich, Zurich, Switzerland; 3 Department DiBEST (Biologia, Ecologia, Scienze della Terra) Unit of Biochemistry and Molecular Biotechnology, University of Calabria, Arcavacata di Rende, Italy; 4 Mechanistic Safety, CMO & Patient Safety, Global Drug Development, Novartis Pharma, Basel, Switzerland

**Keywords:** L-carnitine, cholesterol, methyl-β-cyclodextrin, membrane lipid, membrane transport, proteoliposomes, FBS, fetal bovine serum, jvs, juvenile visceral steatosis, mβcd, methyl-β-cyclodextrin, OCTN2, carnitine/organic cation transporter novel 2, RAMEB, cholesterol-saturated mβcd

## Abstract

The carnitine/organic cation transporter novel 2 (OCTN2) is responsible for the cellular uptake of carnitine in most tissues. Being a transmembrane protein OCTN2 must interact with the surrounding lipid microenvironment to function. Among the main lipid species that constitute eukaryotic cells, cholesterol has highly dynamic levels under a number of physiopathological conditions. This work describes how plasma membrane cholesterol modulates OCTN2 transport of L-carnitine in human embryonic kidney 293 cells overexpressing OCTN2 (OCTN2-HEK293) and in proteoliposomes harboring human OCTN2. We manipulated the cholesterol content of intact cells, assessed by thin layer chromatography, through short exposures to empty and/or cholesterol-saturated methyl-β-cyclodextrin (mβcd), whereas free cholesterol was used to enrich reconstituted proteoliposomes. We measured OCTN2 transport using [^3^H]L-carnitine, and expression levels and localization by surface biotinylation and Western blotting. A 20-min preincubation with mβcd reduced the cellular cholesterol content and inhibited L-carnitine influx by 50% in comparison with controls. Analogously, the insertion of cholesterol in OCTN2-proteoliposomes stimulated L-carnitine uptake in a dose-dependent manner. Carnitine uptake in cells incubated with empty mβcd and cholesterol-saturated mβcd to preserve the cholesterol content was comparable with controls, suggesting that the mβcd effect on OCTN2 was cholesterol dependent. Cholesterol stimulated L-carnitine influx in cells by markedly increasing the affinity for L-carnitine and in proteoliposomes by significantly enhancing the affinity for Na^+^ and, in turn, the L-carnitine maximal transport capacity. Because of the antilipogenic and antioxidant features of L-carnitine, the stimulatory effect of cholesterol on L-carnitine uptake might represent a novel protective effect against lipid-induced toxicity and oxidative stress.

Carnitine is a vitamin-like compound cardinal in the translocation of mid- and long-chain fatty acids from the cytosol into the mitochondrial matrix, where the fatty acid β-oxidation takes place (1, 2). An important experimental model for the comprehension of the role of carnitine in cell metabolism has been and still is the juvenile visceral steatosis (jvs) mouse. Jvs pups are characterized by systemic carnitine deficiency, hepatic steatosis, hypoglycemia and hyperammonemia, and growth retardation (3, 4, 5, 6). This phenotype closely resembles that observed in children and adults diagnosed with primary systemic carnitine deficiency (OMIM212149), a metabolic disorder in which the body cannot properly process fats to produce energy (4). Primary systemic carnitine deficiency in both human and rodents is caused by loss-of-function mutations in the *SLC22A5/Slc22a5* gene encoding for the carnitine/organic cation transporter novel 2 (OCTN2). OCTN2 is a plasma membrane transporter belonging to the SLC22 family and is responsible for the Na^+^-dependent transport of carnitine and its precursor butyrobetaine (7, 8). *In vitro*, OCTN2 also mediates the uptake of a number of drugs (*e.g.*, oxaliplatin, beta-lactams) (9, 10, 11, 12). Experiments in jvs animals indicate that OCTN2 is critical for the intestinal absorption; distribution in liver, skeletal muscle, and heart; and in renal reabsorption of carnitine (3, 4, 5, 6, 13, 14, 15, 16).

In line with the pivotal role of carnitine in lipid metabolism, the OCTN2 expression level is closely linked to lipid homeostasis. The nuclear receptor PPARα, activated by free fatty acids, induces the mRNA expression of OCTN2 in rodents and pig (17, 18, 19, 20, 21, 22). In humans, insulin is associated with an increase in OCTN2 expression and activity in skeletal muscle (23). Overall, OCTN2 induction may represent an adaptive protective mechanism, thereby reducing lipid accumulation and toxicity in cells. Animals fed a high-fat diet display elevated lipids not only in the plasma but also in the cellular membranes of various organs (24, 25, 26). Because of the fine-tuned interaction between transmembrane proteins and the surrounding lipid microenvironment, we hypothesized that the accumulation of lipids in the plasma membrane might as well promote L-carnitine uptake mediated by OCTN2. In particular, plasma membrane cholesterol has been extensively associated with transmembrane protein function (27, 28, 29, 30) through lipid–lipid and/or protein–lipid interactions. By interacting with phospholipids, cholesterol alters the fluidity of the lipid bilayer (31, 32, 33, 34, 35, 36). Cholesterol seems also to bind to transmembrane protein domains known as Cholesterol Recognition/interaction Amino acid Consensus sequences (CRAC and CARC) (37). The aim of the present work is to outline the effects of cholesterol on OCTN2-mediated L-carnitine transport employing intact cells overexpressing the human OCTN2 and proteoliposomes harboring the native OCTN2 extracted from OCTN2-HEK293 cells.

## Results

### Effect of mβcd exposure on cholesterol content and L-carnitine influx in intact cells

**Figure 1:**
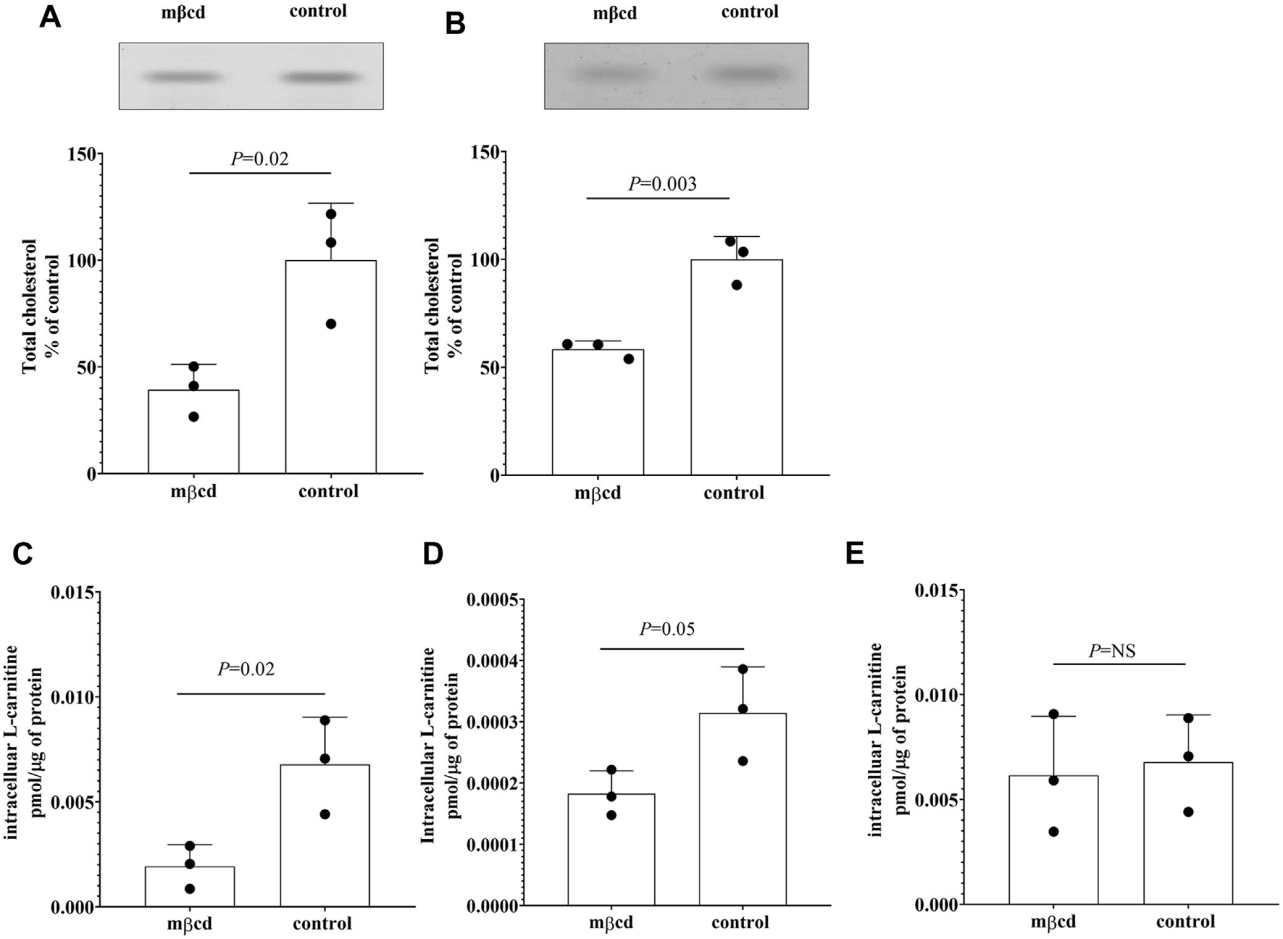
**Cholesterol content and L-carnitine influx in cells overexpressing the human OCTN2.** Representative image of total cellular cholesterol content resolved in a silica plate and quantification of total cholesterol from OCTN2-HEK293 cells (*A*) and OCTN2-Huh-7 cells (*B*) exposed for 20 min to methyl-β-cyclodextrin (mβcd) at the extracellular concentration of 2.5 mM. Data represent the mean ± SD from three independent experiments. Fifteen-second uptake of L-carnitine at the extracellular concentration of 0.5 μM, in OCTN2-HEK293 cells (*C*) and OCTN2-Huh-7 (*D*) cells after 20-min preincubation with mβcd at the extracellular concentration of 2.5 mM. Fifteen-second uptake of L-carnitine at the extracellular concentration of 0.5 μM, in OCTN2-HEK293 cells in the presence of 2.5 mM mβcd (*E*). Uptake data were subtracted of the uptake values in Na^+^-free buffer and expressed as the mean ± SD from three independent experiments. The indicated *p*-values were calculated from unpaired *t* test comparisons. mβcd, methyl-β-cyclodextrin; OCTN2, carnitine/organic cation transporter novel 2.

As previously shown, short exposure to methyl-β-cyclodextrin (mβcd) allows selective scavenging of cholesterol from cells (38). HEK293 cells stably transfected with the coding sequence of the human OCTN2 (Fig. 1*A*) and Human hepatocellular carcinoma cells (Huh-7) cells transiently transfected with the human OCTN2 (Fig. 1*B*) exposed for 20 min to mβcd were characterized by an ∼50% depletion of total cholesterol in comparison with the untreated cells. The impact of cholesterol removal on OCTN2 function was evaluated by assessing the L-carnitine uptake at the nonsaturating extracellular concentration of 0.5 μM in the presence or absence of Na^+^ over 15 s, an interval in which the transport of L-carnitine is linear, reflecting the unidirectional flux of the substrate into the cells (12). As shown in Figure 1*C*, the 20-min preincubation with mβcd significantly reduced the Na^+^-dependent influx of L-carnitine (0.002 ± 0.001 *versus* 0.006 ± 0.003 pmol/μg of protein, *p* = 0.02) in OCTN2-HEK293 cells. In a similar fashion, in OCTN2-Huh-7 cells, the mβcd preincubation reduced the uptake of L-carnitine by ∼50% as compared with that in untreated OCTN2-Huh-7 cells (0.00018 ± 0.00004 *versus* 0.00031 ± 0.00007 pmol/μg of protein, *p* = 0.05) (Fig. 1*D*). A possible direct inhibitory effect of mβcd on L-carnitine OCTN2-mediated transport was ruled out by measuring L-carnitine uptake in the presence of mβcd at the extracellular concentration of 2.5 mM in OCTN2-HEK293 cells. It can be seen that mβcd did not directly interfere with the L-carnitine-OCTN2 interaction (0.006 ± 0.003 *versus* 0.007 ± 0.002 pmol/μg of protein, *p* = NS) (Fig. 1*E*). Because of the relatively low transport rate of L-carnitine measured in OCTN2-Huh-7 cells, thus the low signal-to-noise ratio, the nature of the OCTN2-cholesterol interaction was further investigated exclusively in OCTN2-HEK293 cells. The inhibitory effect of cholesterol depletion L-carnitine uptake mediated by OCTN2 was observed also in OCTN2-HEK293 cells exposed to atorvastatin, an inhibitor of 3-hydroxy-3-methyl-glutaryl-coenzyme A reductase, the rate-limiting enzyme in cholesterol synthesis (Fig. S1).

### Impact of mβcd exposure on OCTN2 localization and lipid raft integrity

**Figure 2:**
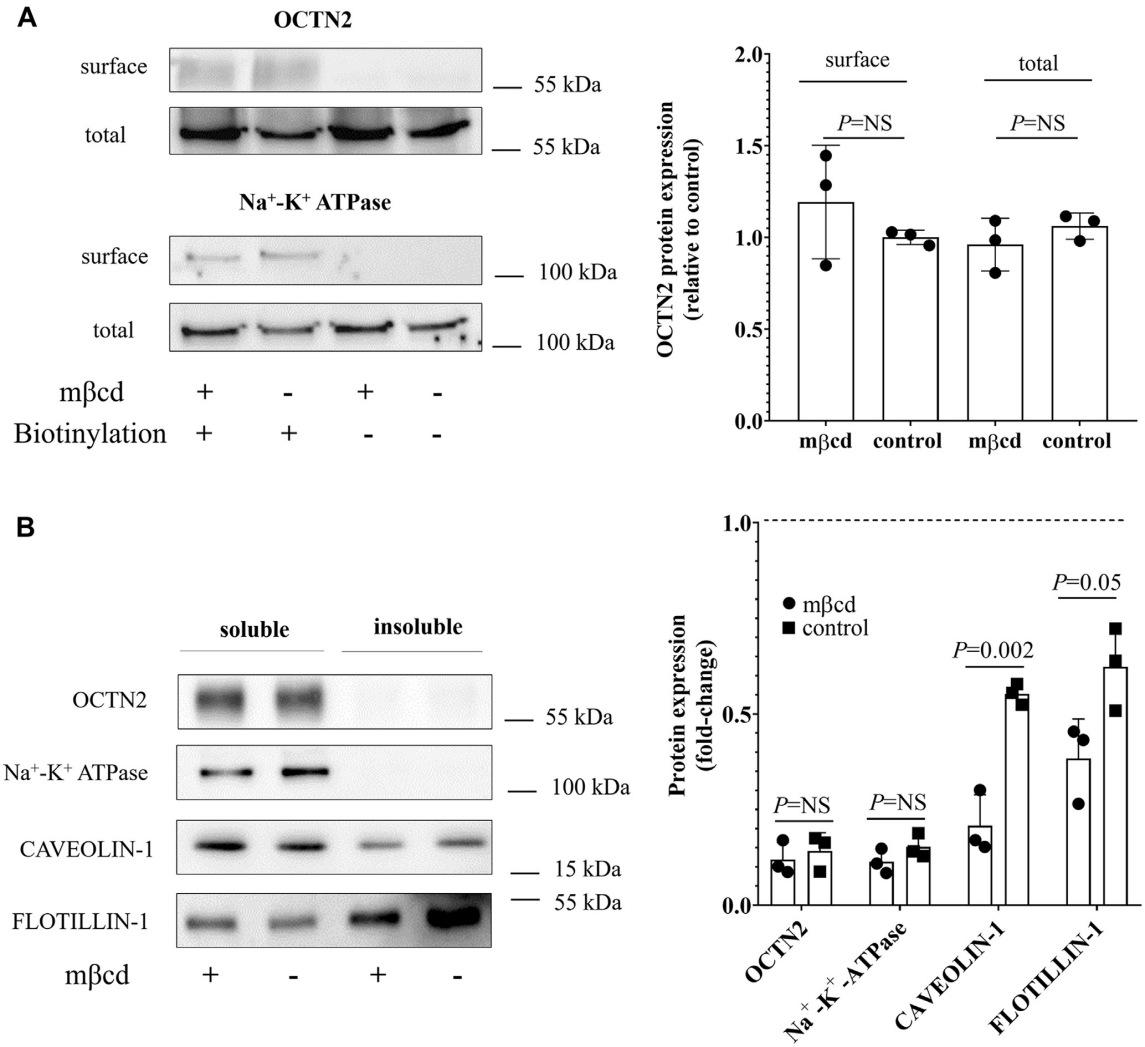
**Plasma membrane localization of OCTN2.** Representative Western blot and relative quantification from three independent experiments of OCTN2 and Na^+^/K^+^ ATPase expression biotinylated at the cell surface of OCTN2-HEK293 cells exposed for 20 min to mβcd at the extracellular concentration of 2.5 mM. The indicated *p*-values were calculated from unpaired *t* test comparisons (*A*). Representative Western blot and relative quantification of the insoluble fractions (rafts) from three independent experiments of OCTN2, Na^+^/K^+^ ATPase, CAVEOLIN-1, and FLOTILIN-1 expression biotinylated at the cell surface of OCTN2-HEK293 cells exposed for 20 min to mβcd at the extracellular concentration of 2.5 mM. Na^+^/K^+^ ATPase was used as the nonraft marker, and CAVEOLIN-1 and FLOTILIN-1 were used as raft markers. The indicated *p*-values were calculated from unpaired *t* test comparisons (*B*). mβcd, methyl-β-cyclodextrin; OCTN2, carnitine/organic cation transporter novel 2.

Surface labeling coupled to Western blotting was employed to assess the OCTN2 expression level in OCTN2-HEK293 cells upon exposure to mβcd. As shown in Figure 2*A*, the plasma membrane OCTN2 protein level was comparable in control and mβcd-treated cells.

Cholesterol distribution in the membrane is heterogeneous, concentrating in cholesterol-rich and sphingomyelin-rich membrane domains (membrane rafts). Several studies have shown that cholesterol depletion results in the spatial reorganization of membrane proteins because of the shattering of the cholesterol-enriched domains (39). In addition, studies in rat astrocytes demonstrated that plasma membrane Octn2 localizes primarily in lipid rafts, where it directly interacts with caveolin-1, a membrane protein enriched in lipid rafts (40). In order to examine whether OCTN2 localized in membrane rafts and to what extent OCTN2 membrane localization was affected by the treatment with mβcd, the surface expression level of OCTN2 was assessed in the raft (insoluble) and nonraft (soluble) fractions of OCTN2-HEK293 cells. Figure 2*B* shows that, upon cholesterol depletion, there was a significant disruption of the raft fraction, as shown by the redistribution of CAVEOLIN-1 and FLOTILLIN-1 from the insoluble (rafts) to the soluble fraction (nonrafts). Moreover, it can be seen that both Na^+^/K^+^ ATPase, a known nonraft marker (41), and OCTN2, were almost exclusively detected in the soluble fraction (nonrafts), suggesting that OCTN2 did not cluster in the lipid rafts.

### Impact of mβcd exposure on membrane integrity and membrane potential

**Figure 3:**
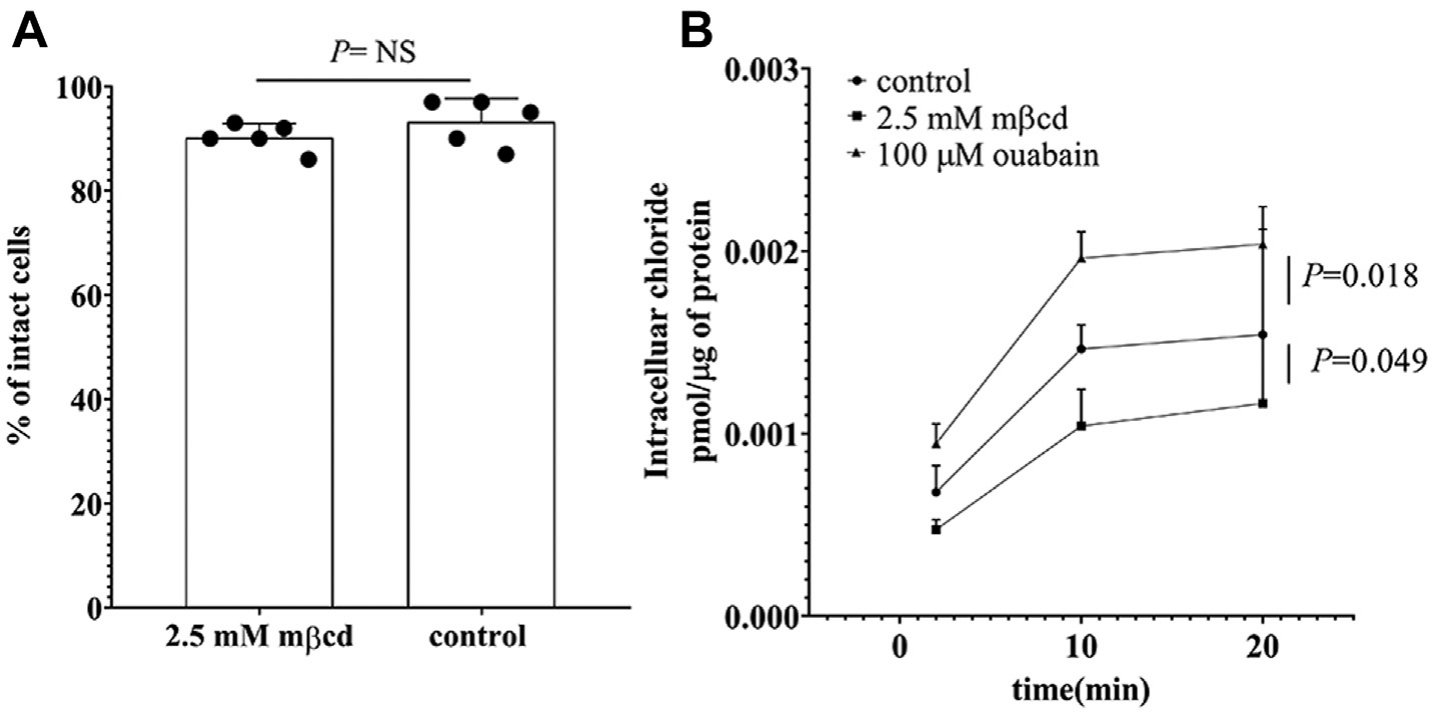
**Membrane integrity and chloride distribution across the plasma membrane of OCTN2-HEK293 cells.** Percentage of cells impermeable to Trypan Blue dye. Data represent the mean ± SD from five independent experiments (*A*). Intracellular chloride content as a function of the time in OCTN2-HEK293 cells after 20-min preincubation with the indicated conditions. Results are expressed as the mean ± SD from three independent experiments (*B*). Multiple comparisons were performed using one-way ANOVA test followed by Tukey’s test. Significant differences between the means of two groups were calculated from unpaired *t* test comparisons. mβcd, methyl-β-cyclodextrin; OCTN2, carnitine/organic cation transporter novel 2.

Plasma membrane leaking and/or the inhibition of the Na^+^/K^+^ ATPase might as well contribute to the reduced uptake of L-carnitine observed upon preincubation to mβcd (7, 42). To assess the plasma membrane integrity, OCTN2-HEK293 cells were exposed for 20 min to mβcd and then mixed with the Trypan blue solution for cell counting. Figure 3*A* shows that the fractions of control and mβcd-treated cells impermeable to Trypan blue were comparable, indicating that mβcd preincubation did not alter the plasma membrane integrity. The activity of the Na^+^/K^+^ ATPase is critical to sustain the Na^+^ inward gradient, hence to the L-carnitine OCTN2-mediated transport. A reduction in the Na^+^/K^+^ ATPase activity or an outward leaking of Na^+^ would result in a reduced Na^+^ inward gradient and, in turn, in membrane potential depolarization. As chloride distribution has been shown to correlate well with the cell membrane potential (43, 44, 45, 46), the impact of mβcd preincubation on plasma membrane potential was assessed by monitoring the distribution of chloride ions across the plasma membrane. Figure 3*B* shows that, at equilibrium, the intracellular level of chloride ions was increased by a 20-min exposure to ouabain, which inhibits the Na^+^/K^+^ ATPase, causing the partial depolarization of the plasma membrane (*p* = 0.018). Conversely, the preincubation with mβcd slightly, albeit significantly, hyperpolarized the plasma membrane as reflected by the lower intracellular chloride ion concentration (*p* = 0.049).

### Effect of exogenous cholesterol on total cholesterol levels and OCTN2-mediated L-carnitine transport

**Figure 4:**
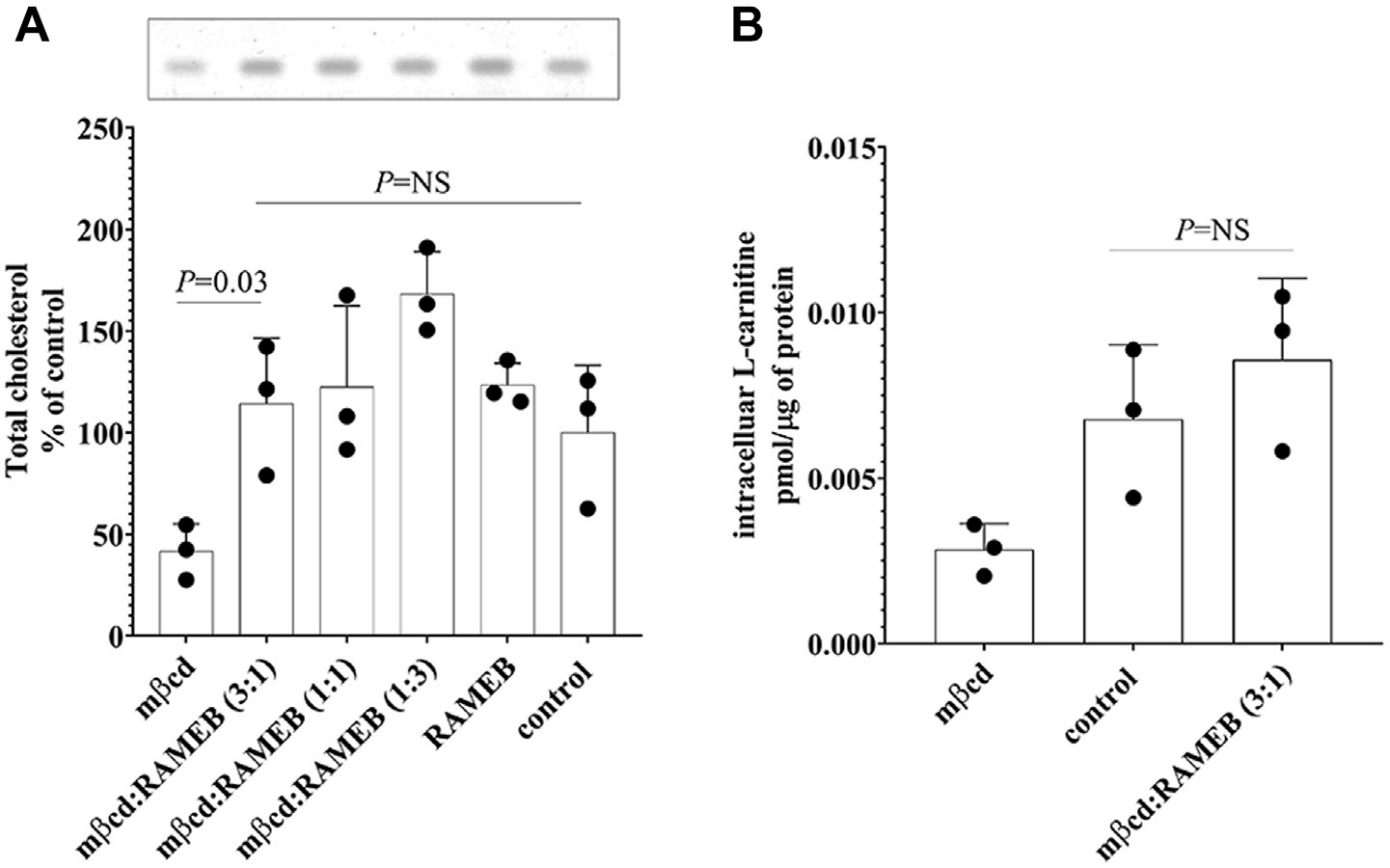
**Cholesterol content and OCTN2-mediated carnitine influx after exposure to exogenous cholesterol.** Representative image of total cellular cholesterol resolved in a silica plate and quantification from OCTN2-HEK293 cells treated for 20 min with the indicated conditions. Data represent the mean ± SD from three independent experiments (*A*). Fifteen-second uptake of L-carnitine in OCTN2-HEK293 cells after 20-min preincubation with mβcd or mβcd:RAMEB (3:1) mixture. Results were subtracted of the uptake values in Na^+^-free buffer and expressed as the mean ± SD from three independent experiments (*B).* The indicated *p*-values were calculated using one-way ANOVA test followed by Tukey’s multiple comparison test. mβcd, methyl-β-cyclodextrin; OCTN2, carnitine/organic cation transporter novel 2; RAMEB, cholesterol-saturated mβcd.

To assess whether the effect of mβcd on OCTN2-mediated L-carnitine influx was cholesterol dependent, “cholesterol-matched” experiments were performed. It can be seen that OCTN2-HEK293 cells exposed for 20 min to varying ratios of empty mβcd:cholesterol-saturated mβcd (RAMEB) mixtures showed similar total cholesterol content to that of control cells (Fig. 4*A*). The 3:1 mβcd:RAMEB ratio was used for assessing L-carnitine transport. OCTN2-mediated L-carnitine influx after preincubation with mβcd:RAMEB (3:1) was comparable with that in control cells (0.009 ± 0.002 *versus* 0.007 ± 0.002 pmol/μg of protein, *p* = NS), indicating that the mβcd effect on OCTN2-mediated L-carnitine transport is cholesterol dependent (Fig. 4*B*).

### Effect of cholesterol incorporation on L-carnitine OCTN2-mediated transport in reconstituted proteoliposomes

**Figure 5:**
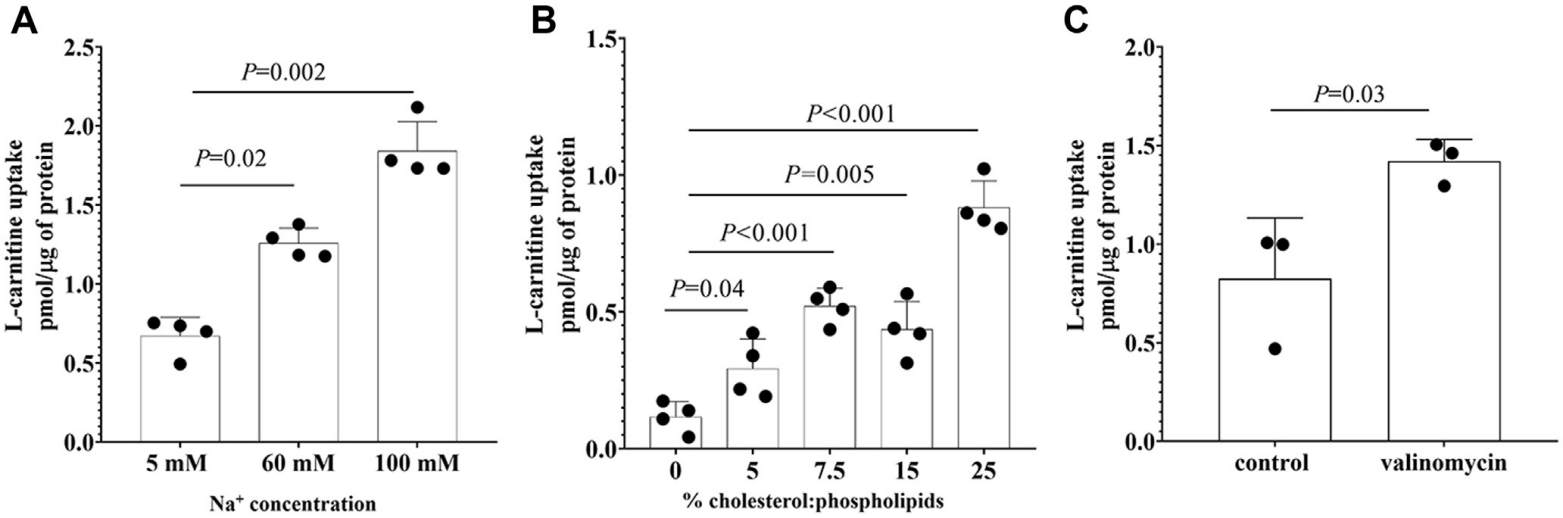
**L-carnitine uptake in proteoliposomes harboring the human OCTN2.** One-minute transport of 80 μM L-carnitine at increasing extracellular concentrations of Na^+^. Results are expressed as the mean ± SD from four independent experiments (*A*). One-minute transport of 80 μM L-carnitine in the presence of 60 mM Na^+^ in proteoliposomes containing the indicated nominal cholesterol:phospholipid ratio. Data represent the mean ± SD from four independent experiments (*B*). One-minute transport of 80 μM L-carnitine in the presence of 60 mM Na^+^ in proteoliposomes loaded with 25% cholesterol and treated with valinomycin. Data represent the mean ± SD from three independent experiments (*C*). Multiple comparisons were performed using one-way ANOVA test followed by Tukey’s test. Significant differences between the means of two groups were calculated from unpaired *t* test comparisons. OCTN2, carnitine/organic cation transporter novel 2.

The study of the effect of cholesterol on OCTN2 activity in intact cells was limited by the resistance of the OCTN2-HEK293 cells to cholesterol overloading (Fig. 4*A*) and by the deleterious effect of more extensive cholesterol depletion, which is likely to jeopardize cell viability. Hence, to better characterize the dependence of OCTN2 transport activity on cholesterol content, OCTN2 protein was extracted from OCTN2-HEK293 cells and reconstituted in egg yolk phospholipid-liposomes. Figure 5*A*, which illustrates the uptake of L-carnitine in the presence of increasing concentration of Na^+^, demonstrates that the proteoliposomes harboring OCTN2 were functional. In line with the results obtained in intact cells, the transport of L-carnitine was stimulated by the incorporation of exogenous cholesterol and such effect appeared to be dose dependent (Fig. 5*B*). Finally, the transport of L-carnitine was stimulated by a potassium diffusion membrane potential induced in the presence of valinomycin (Fig. 5*C*). Taken together, the data indicate that cholesterol stimulated OCTN2 transport activity in proteoliposomes as well and that the stimulatory effect on L-carnitine uptake was the result of the interaction with the OCTN2 protein and not a secondary effect of the Na^+^/K^+^ pump.

### Effect of cholesterol content on OCTN2-mediated influx kinetics of L-carnitine

**Figure 6:**
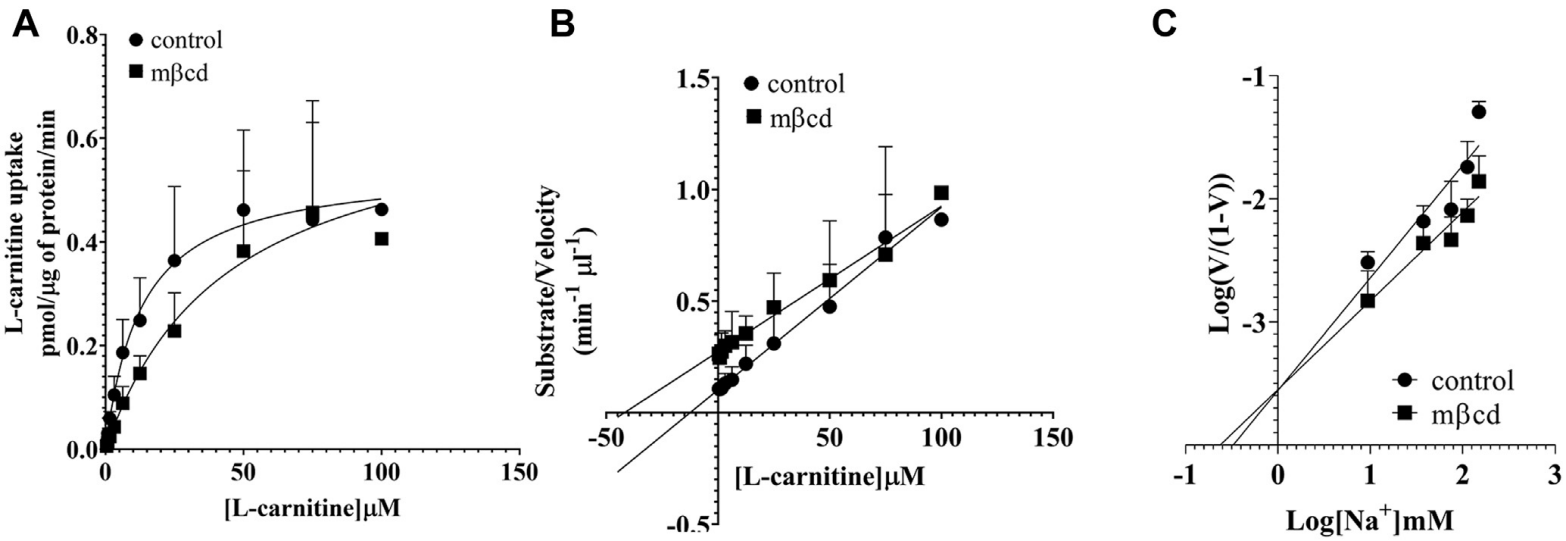
**Kinetic analysis of L-carnitine influx in intact cells.** Initial uptake of L-carnitine was assessed in OCTN2-HEK293 cells at increasing extracellular concentrations of L-carnitine (*A*–*B*) or Na^+^ (*C*), after 20-min preincubation with mβcd at the extracellular concentration of 2.5 mM. Data were corrected for uptake in Na^+^-free buffer and expressed as the mean ± SD from at least three independent experiments. The line is best fit to the Michaelis–Menten equation (V = *V*_max_[S]/(*K*_m_ + [S])) (*A*). A Hanes–Woolf (*B*) and a Hill (*C*) plot of the data. mβcd, methyl-β-cyclodextrin; OCTN2, carnitine/organic cation transporter novel 2.

**Figure 7:**
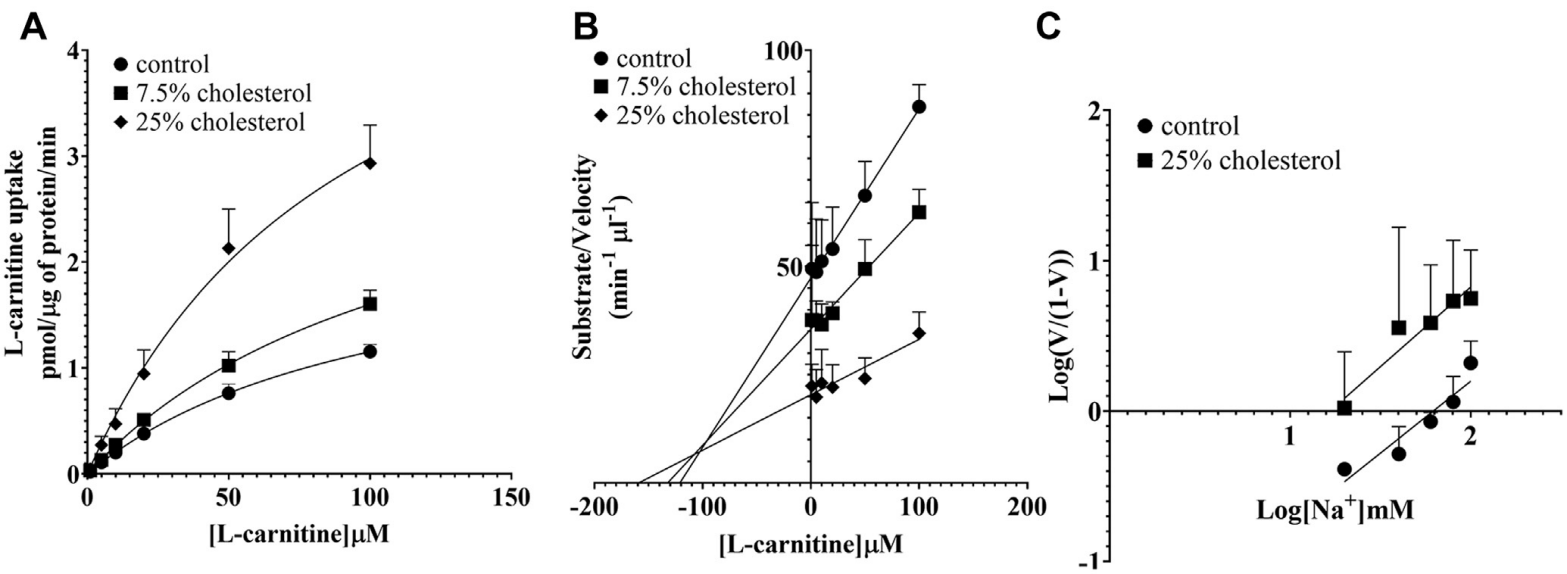
**Kinetic analysis of L-carnitine influx in proteoliposomes.** Initial uptake of L-carnitine was assessed in proteoliposomes harboring the human OCTN2 at increasing extracellular concentrations of L-carnitine (*A*–*B*) or Na^+^ (*C*). Data were corrected for uptake in Na^+^-free buffer and expressed as the mean ± SD from at least three independent experiments. The line is best fit to the Michaelis–Menten equation (V = *V*_max_[S]/(*K*_m_ + [S])) (*A*). A Hanes-Woolf (*B*) and a Hill (*C*) plot of the data. OCTN2, carnitine/organic cation transporter novel 2.

To understand the nature of cholesterol-dependent stimulation of OCTN2-mediated L-carnitine transport, the influx kinetics of L-carnitine was measured in OCTN2-HEK293 cells after 20-min preincubation with mβcd at the extracellular concentration of 2.5 mM. Cholesterol depletion increased the *K*_m_ of OCTN2 for L-carnitine (19.9 ± 7.79 *versus* 76.9 ± 26.5 μM, *p* = 0.02) with a slight, albeit significant, stimulation of the maximal capacity of the transport (0.63 ± 0.13 *versus* 0.92 ± 0.12 pmol/μg of protein/min, *p* = 0.04) (Fig. 6*A*). Although the kinetic values obtained from the Hanes–Woolf transformation (Fig. 6*B*) were comparable with those calculated from the hyperbola derived from the Michaelis–Menten equation, it is noteworthy that, in the latter, the influx *K*_m_ for L-carnitine upon treatment with mβcd might be overestimated as saturation of the transport reaction could not be reached. Kinetic parameters of L-carnitine transport mediated by OCTN2 are summarized in Table 1. To understand whether the maximal capacity augmentation upon cholesterol depletion was because of an increased affinity for the Na^+^, L-carnitine influx was measured at increasing extracellular concentrations of Na^+^. Figure 6*C* shows that the regression lines of the L-carnitine influx as a function of the extracellular concentration of Na^+^ in control and mβcd-treated cells nearly intersected on the *x*-axis, indicating comparable *K*_m_ for the Na^+^ (3.60 ± 0.26 *versus* 5.13 ± 0.70 mM, *p* = NS). Kinetic analysis in proteoliposomes showed that, upon cholesterol insertion the *V*_max_ increased in a dose-dependent manner with no changes in the affinity toward L-carnitine. The *V*_max_ of L-carnitine influx in the presence of 25% cholesterol (25 μg cholesterol/mg of lipids) was higher than that in control proteoliposomes (6.15 ± 1.34 *versus* 2.47 ± 0.3 pmol/μg of protein/min, *p* = 0.0003) (Fig. 7, *A*–*B*, Table 1). To understand whether the effect of cholesterol insertion on the *V*_max_ was secondary to a change in the affinity for the binding of Na^+^ to OCTN2, the influx of L-carnitine was measured in reconstituted proteoliposomes titrating the concentration of Na^+^ in the transport buffer. It can be seen that in cholesterol-loaded proteoliposomes the affinity for Na^+^ was ∼4 times stronger than that in control proteoliposomes (*p* = 0.03), suggesting that the cholesterol loading did change the affinity of OCTN2 for the Na^+^ (Fig. 7*C*).

**Table 1:** Kinetic parameters of L-carnitine transport rate mediated by OCTN2

Analysis	Treatment	*K*_m_	*p*-value	*V*_max_	*p*-value
Intact cells					
Michaelis–Menten	Control	19.9 ± 7.79	0.02	0.63 ± 0.13	0.04
	2.5 mM mβcd	76.9 ± 26.5		0.92 ± 0.12	
Hanes–Woolf	Control	17.3 ± 4.24	0.003	0.62 ± 0.14	NS
	2.5 mM mβcd	53.3 ± 8.31		0.78 ± 0.23	
Proteoliposomes					
Michaelis–Menten	Control	115 ± 38.3	NS	2.47 ± 0.30	0.0003
	25% Cholesterol	108 ± 48.3		6.15 ± 1.34	
Hanes–Woolf	Control	126 ± 46.5	NS	2.60 ± 0.40	0.008
	25% Cholesterol	131 ± 34.2		8.68 ± 3.86	

*K*_m_ values are expressed in μM.

*V*_max_ values are expressed as pmol μg^−1^ min^−1^.

## Discussion

OCTN2 transports L-carnitine in a Na^+^-dependent manner with the binding to the Na^+^ and its inward gradient across the plasma membrane dictating the cycling of the protein and, in turn, the transport capacity (7, 14). Here, we show that the transport of L-carnitine mediated by the human OCTN2 is markedly stimulated by the presence of cholesterol in the plasma membrane. In both the experimental models employed, OCTN2-HEK293 cells and proteoliposomes harboring OCTN2, cholesterol stimulates the catalytic efficiency, calculated as the ratio between the *V*_max_ and the *K*_m_, of OCTN2-mediated L-carnitine uptake by ∼3-fold. Yet, the nature of the stimulatory effect varies depending on the experimental model used. When cholesterol is partially removed from living cells, the affinity of OCTN2 for L-carnitine falls, whereas the binding affinity to the Na^+^ does not change. When cholesterol is added to proteoliposomes harboring OCTN2 extracted from OCTN2-HEK293 cells, the stimulation is strictly dependent on the enhanced binding of OCTN2 to the Na^+^. It is possible that the reduced affinity for L-carnitine upon depletion of cholesterol measured in intact HEK293 cells, but not in proteoliposomes, is the result of the disruption of heterogeneous tertiary complexes of OCTN2–cholesterol–phospholipids that might be underrepresented in the liposomes, which are essentially constituted of phosphatidylcholine. Although sparse, studies demonstrated that each phospholipid exhibits unique changes in enthalpy, transition temperature, and cooperativity within the membrane with progressive increases in cholesterol content (47, 48, 49). It is also interesting to notice that, in HEK293 cells, OCTN2 does not localize in cholesterol/sphingolipid microdomains (lipid rafts), suggesting that, in this particular setting, the loss of integrity of the lipid rafts upon the treatment with mβcd may not play a role in the loss of affinity of OCTN2 for L-carnitine. These results clash with previous findings describing that in rat astrocytes Octn2 localizes primarily in lipid rafts upon activation of the protein kinase C (PKC) (40). It seems plausible that the cascade induced by PKC activation is important not only for the trafficking of OCTN2 to the membrane but also for its spatial organization between raft and nonraft domains.

Although cholesterol depletion from OCTN2-HEK293 cells does not affect the binding of Na^+^ to the carrier, a slight increase in the L-carnitine transport capacity of OCTN2 can be noticed, suggesting that the cholesterol depletion might still alter the inward Na^+^ gradient across the plasma membrane. Indeed, when compared with control cells, cholesterol-depleted cells are characterized by a plasma membrane hyperpolarization, which is consistent with a greater inward Na^+^ gradient resulting perhaps from an enhanced activity of the Na^+^-K^+^ ATPase. The dependence of the Na^+^-K^+^ ATPase enzyme on membrane cholesterol level is highly controversial with biphasic, stimulatory, or inhibitory effects observed, depending on the experimental model employed (50, 51, 52, 53, 54, 55, 56, 57), and on the method used for the cholesterol depletion (58, 59, 60). Whatever the effect is, it is likely to reflect the balance between (i) the direct impact of the cholesterol depletion on the enzymatic activity and (ii) the secondary effects ascribable to the increased membrane permeability to ions such as proton and Na^+^, upon cholesterol depletion (61).

Plasma membrane cholesterol has been shown to have a biphasic effect on transporters’ activity. Examples are the Na^+^-K^+^ ATPase and the amino acid transporter ASCT2. In human red blood cells, the Na^+^-K^+^ ATPase activity is stimulated by a 5% to 25% reduction in the cholesterol level but inhibited by a 35% to 50% depletion (50). In a similar vein, the amino acid transporter ASCT2 has an optimum of activity in 7.5% cholesterol proteoliposomes; however, at higher contents of cholesterol, the transport activity is inhibited (62). Such a biphasic response supports the idea of an “optimum” membrane cholesterol for maximal activity of these membrane transport systems. This does not appear to be the case for OCTN2. The effect of cholesterol on the influx rate of L-carnitine in proteoliposomes is dose dependent across the range of cholesterol content employed, very much like in the case of the organic cation transporter novel 1 (OCTN1), whose activity is stimulated by cholesteryl hemisuccinate in a dose-dependent manner solely through an increase of the transport capacity (63). Cholesterol has been shown to alter also the transport activity of rat Oct1 and human OCT2 in yet another way. Cell-free-expressed rat Oct1 reconstituted in nanodiscs or proteoliposomes displays the allosteric high-affinity binding site only in the context of certain lipid microenvironment (*e.g.*, the presence of cholesterol) (64). Analogously, the allosterism of the human OCT2 is abolished when cholesterol is removed from the cells (38).

Elevated cellular cholesterol levels have been found in erythrocytes, adipocytes, and skeletal muscle cells of animals fed a high-fat diet (24, 25, 26); in aging tissues (65, 66, 67, 68, 69); and within the context of several acute and chronic disorders including acute kidney injury (70), liver disease (71), and Alzheimer’s (72). It has been suggested that the raising of plasma membrane cholesterol level represents a protective mechanism that the cell employs under acute or chronic stress (73, 74). Because of the critical role of OCTN2 in L-carnitine uptake in most tissues (75), it is plausible that such elevation in cellular cholesterol might be coupled to a greater intracellular accumulation of L-carnitine, which can exert multiple protective effect. By increasing the rate of β-oxidation of medium- and long-chain fatty acids, L-carnitine can buffer the harmful effect of lipid accumulation, which, when coupled to oxidative stress, leads to lipid peroxidation, cell death, inflammation, and fibrosis (1, 76, 77, 78). Moreover, L-carnitine can directly prevent the opening of the mitochondrial transition pore (79, 80), which leads to mitochondrial depolarization, inhibition of cellular respiration, ATP depletion, and cell death (81).

## Experimental procedures

### Reagents

Dulbecco's modified Eagle’s medium, RPMI 1640 medium, penicillin/streptomycin, and Geneticin G-418 were purchased from ThermoFisher Scientific (Waltham, MA, USA). Biowest fetal bovine serum (FBS) was provided by VWR (Dietikon, CH). Poly-D-lysine was purchased from Corning (Bedford, MA, USA). L-[methyl-^3^H]carnitine hydrochloride ([^3^H]L-carnitine, specific activity: 81.0 Ci/mmol) and ^36^Chloride (specific activity: 10 mCi/g) were synthesized by Amersham Life Sciences (Piscataway, NJ, USA); [4-^14^C]-cholesterol ([^14^C]cholesterol, specific activity: 50.8 mCi/mmol) was purchased from PerkinElmer (Boston, MA, USA). Nonlabeled L-carnitine, methyl-β-cyclodextrin (mβcd), and nonlabeled cholesterol were provided by Sigma-Aldrich (St. Louis, MO, USA). Cholesterol-saturated mβcd (RAMEB) was provided by CycloLab Ltd (Budapest, Hungary).

### Cell culture

HEK293 cells stably transfected with the coding sequence of human OCTN2 (OCTN2-HEK293) were previously characterized (14, 82). Cells were maintained in Dulbecco's modified Eagle's medium supplemented with 10% FBS, 100 units/ml penicillin, 100 μg/ml streptomycin, with Geneticin G-418 at a concentration of 600 μg/ml. Human hepatocellular carcinoma cells (Huh-7) were grown in RPMI 1640 medium supplemented with 10% FBS, 100 units/ml penicillin, and 100 μg/ml streptomycin.

### Transient transfection of Huh-7 cells

For lipid extraction and transport assay, 3 × 10^5^ cells were seeded onto 6-well plates and 3.5-cm dishes, respectively. After 2 days, cells were transfected with pcDNA3.1(+) plasmid containing the coding sequence of the human OCTN2 (2 μg/well) with Lipofectamine 2000 (Invitrogen, Carlsbad, CA, USA) in serum-free and antibiotic-free medium. Experiments were performed 2 days after transfection.

### OCTN2 reconstitution in proteoliposomes

HEK293 cells with stable overexpression of OCTN2 were harvested and resuspended in lysis buffer containing 1% (w/v) Triton-100, 20 mM Hepes/NaOH pH 7.5, and protease inhibitors. After incubation on ice for 30 min, the lysate was centrifuged at 12,000*g*_av_ for 15 min at 4 °C. The supernatant was reconstituted by removing the detergent from mixed micelles containing detergent, protein, and phospholipids. The composition of the initial mixture was 100 μg of total proteins, 75 μl of 10% Triton X-100, 110 μl of 10% egg yolk phospholipids in the form of sonicated liposomes, and 20 mM Hepes (pH 7.5) in a final volume of 700 μl. The detergent was removed by incubating the mixture with 0.55 g Amberlite XAD-4 for 60 min under stirring at 22 °C (83).

### Assessment of plasma membrane integrity

OCTN2-HEK293 cells were seeded onto 6-well plates coated with 0.1 mg/ml poly-D-lysine at a density of 3 × 10^5^ cells/well. After 72 h, the cells were washed with phosphate-buffered saline (PBS) and then treated for 20 min at 37 °C with mβcd dissolved in Na^+^-containing transport buffer (116.4 mM NaCl, 5.3 mM KCl, 1 mM NaH_2_PO_4_, 0.8 mM MgSO_4_, 5.5 mM D-glucose, and 20 mM Hepes/KOH, pH 7.4). Finally, the cells were washed with PBS and resuspended in a 0.02% Trypan Blue solution for cell counting.

### Uptake assay in intact cells

OCTN2-HEK293 cells were seeded onto 3.5-cm dishes coated with 0.1 mg/ml poly-D-lysine at the density of 3 × 10^5^ cells/well. After 72 h, the cells were washed twice with Na^+^-containing transport buffer. The cells were exposed to Na^+^-containing transport buffer spiked with [^3^H]L-carnitine or ^36^chloride. Uptake was stopped by quick aspiration and extensive washing with ice-cold Na^+^-containing transport buffer. Cells were solubilized with 1 ml of 1% (w/v) Triton X-100 solution. A total of 500 μl of the lysate was transferred in scintillation vials to assess the intracellular radioactivity. A 25-μl aliquot was used for protein determination by the bicinchoninic acid protein assay (Interchim, Montluçon Cedex, France). For determination of OCTN2-independent uptake of L-carnitine, the uptake was measured in Na^+^-free transport buffer, in which the Na^+^ was replaced with the monocation choline. This value was subtracted from the uptake in Na^+^-containing transport buffer to determine the L-carnitine OCTN2-mediated transport. The uptake of L-carnitine in Na^+^-free transport buffer accounts for ∼15% of the uptake in transport buffer containing Na^+^. The uptake of L-carnitine in OCTN2-Huh-7 cells was assessed 48 h after transfection. In this case, the uptake of L-carnitine in Na^+^-free transport buffer accounts for ∼70% of the uptake in Na^+^-containing buffer.

### Uptake assay in proteoliposomes

A 550-μl aliquot of proteoliposomes was filtered through a Sephadex G-75 column (0.7 cm diameter × 15 cm height). One hundred 100-μl aliquots of eluate were transferred to reaction vessels and used for transport measurement. Uptake assay was performed at 25 °C and started by adding 50 μM L-carnitine spiked with [^3^H]L-carnitine to proteoliposomes. The transport was stopped by washing the proteoliposomes by chromatography using pirces columns (0.6 cm diameter × 8 cm height) containing Sephadex G-75. Intraliposomal radioactivity was measured as reported (83).

### Lipid extraction

Lipid extraction was performed with a standard chloroform/methanol method (84). Cells were seeded at a density of 3 × 10^5^ cells/well onto 6-well plates coated with 0.1 mg/ml poly-D-lysine. After 72 h, the cells were harvested and resuspended in 1 ml of PBS in glass tubes. A total of 100 μl was lysed with 400 μl of 1% (w/v) Triton X-100 for bicinchoninic acid protein determination. The remaining cell suspension was mixed with 3 ml of chloroform:methanol (2:1) solution spiked with [^14^C]cholesterol, serving as internal standard. After 20 min in shaking, samples were centrifuged for 5 min at 1500*g*_av_ for phase separation. The upper phase and interphase were discarded, and the lower phase containing the lipid fraction was dried under a nitrogen flux at 30 °C. Finally, the lipid pellet was resuspended in 300 μl of ice-cold chloroform. A 50-μlaliquot was used for assessing radioactivity by liquid scintillation counting.

### Thin layer chromatography

For the analysis of the cholesterol content, aliquots from the extracted lipids were loaded on HPTLC Silica gel 60 plates with a concentrating zone (Merck KGaA, Darmstadt, Germany) using an automated Camag TLC sampler ATS4 and separated by one-dimensional thin layer chromatography (TLC). Cholesterol was resolved in 62.4% n-hexane, 18.3% n-heptane, 18.3% diethyl ether, and 1% acetic acid. Staining was performed in 9.6% orthophosphoric acid (v/v) and 3% copper acetate (w/v), and then the plate was dried at 120 to 130 °C for 30 min. Bands were scanned at 366 nm, and absolute quantification was performed from a serial dilution of cholesterol resolved in parallel. The values were then normalized for the respective [^14^C]cholesterol levels and the protein content.

### Cell surface labeling

OCTN2-HEK293 cells were seeded onto a 6-well plate coated with 0.1 mg/ml poly-D-lysine. After 72 h, cells were washed twice with PBS and incubated on ice with EZ-Link Sulfo-NHS-LC-biotin (ThermoFisher Scientific), dissolved in PBS at the final concentration of 1 mg/ml. After 1-h incubation, cells were washed twice with PBS and then incubated for 30 min with hypotonic buffer (0.5 mM Na_2_HPO_4_ and 0.1 mM EDTA at pH 7.0) containing protease inhibitors (Roche Diagnostics GmbH, Mannheim, Germany). Cells were harvested by centrifugation at 16,000*g*_av_ for 15 min at 4 °C, rotated for 2 h in 200 μl of lysis buffer (50 mM Tris base, 150 mM NaCl, 1% Nonidet P-40, 0.5% sodium deoxycholate, pH 7.4) containing protease inhibitors and then spun down at 16,000*g*_av_ at 4 °C for 15 min. A portion of the supernatant was used for total lysate analysis. The remaining portion was incubated with streptavidin–agarose beads (50 μl/sample) (ThermoFisher Scientific) in rotation, overnight at 4 °C. To stop the incubation, the beads were washed twice in lysis buffer and twice in lysis buffer supplemented with 2% (w/v) SDS. After the final wash, the proteins bound to the beads were stripped by incubation at 95 °C for 5 min in Laemmli buffer containing 1.5% (w/v) dithiothreitol.

### Isolation of detergent-resistant membranes

OCTN2-HEK293 cells were seeded onto 6-well plates coated with 0.1 mg/ml poly-D-lysine. After 72 h, cells were washed twice with PBS and incubated on ice with EZ-Link Sulfo-NHS-LC-biotin (ThermoFisher Scientific), dissolved in PBS at the final concentration of 1 mg/ml. After 1-h incubation, cells were washed twice with PBS, harvested by centrifugation at 16,000*g*_av_ for 15 min at 4 °C and incubated on ice with 1% (w/v) Triton-X 100 in PBS containing protease inhibitors. After 15 min, the lysate was spun down at 16,000*g*_av_ for 15 min at 4 °C, the supernatant (soluble fraction) was collected, and the pellet (insoluble fraction) was rotated for 2 h in 200 μl of lysis buffer (50 mM Tris base, 150 mM NaCl, 1% Nonidet P-40, 0.5% sodium deoxycholate, pH 7.4) containing protease inhibitors. The biotinylated proteins were pulled down as described above.

### Immunoblotting

Protein samples were loaded onto 8% polyacrylamide gels. Proteins were transferred onto nitrocellulose membranes (GE HealthCare, Piscataway, NJ, USA). The membranes were blocked with 5% (w/v) nonfat dry milk in PBS supplemented with 0.1% (w/v) Tween 20 and incubated overnight at 4 °C with anti-OCTN2 (NBP2-57222, Novusbio, Littleton, CO, USA), followed by probing with a horseradish peroxidase–conjugated anti-rabbit IgG antibody (G21234, ThermoFisher Scientific). As a control, the sample blots were probed with anti-Na^+^/K^+^ ATPase (ab7671, Abcam, Cambridge, UK), anti-CAVEOLIN-1 (610060, BD biosciences, San Jose, CA, USA) and anti-FLOTILLIN-1 (610821, BD biosciences). Blots were developed with SuperSignal West Femto Maximum Sensitivity Substrate (ThermoFischer Scientific) and Fusion FX7 (Vilber Lourmat, Eberhardzell, Germany).

### Statistical analysis

Statistical comparisons were performed using GraphPad Prism (version 8.0 for Windows, GraphPad Software). Comparisons between two groups were performed with the two-tailed Student's unpaired *t* test. For multiple comparisons, one-way ANOVA followed by Tukey’s post hoc test was employed.

## Data availability

All data are contained within the article and the supplementary information available online.

## Conflict of interest

The authors declare no conflicts of interest in regards to this manuscript.
