# Supplementary material for: Cholesterol stimulates the cellular uptake of L-carnitine by the carnitine/organic cation transporter novel 2 (OCTN2)
Source: J Biol Chem. 2021 Jan 5;296:100204. doi: 10.1074/jbc.RA120.015175 (PMC7948396; doi:10.1074/jbc.RA120.015175)
Supplement: Figure S1 [file mmc1.pdf]

Cholesterol stimulates the cellular uptake of L-carnitine mediated by the carnitine/organic cation transporter novel 2 (OCTN2)

Lu Zhang, Ting Gui, Lara Console, Mariafrancesca Scalise, Cesare Indiveri, Stephanie Hausler, Gerd A Kullak-Ublick, Zhibo Gai, Michele Visentin

Experimental procedures

Reagents

Dulbecco's Modified Eagle Medium (DMEM), penicillin/streptomycin and Geneticin G-418 were purchased from ThermoFisher Scientific (Waltham, MA). Biowest fetal bovine serum (FBS) was provided by VWR (Dietikon, CH). Poly-D-lysine was purchased from Corning (Bedford, MA). L-[methyl-^{3}H]carnitine hydrochloride ([^{3}H]L-carnitine, specific activity: 81.0 Ci/mmol) was synthesized by Amersham Life Sciences (Piscataway, NJ), [4-^{14}C]-cholesterol ([^{14}C]cholesterol, specific activity: 50.8 mCi/mmol) was purchased from PerkinElmer (Boston, MA). Atorvastatin, non-labeled L-carnitine and non-labeled cholesterol were provided by Sigma-Aldrich (St.Louis, MO).

Cell culture

HEK293 cells stably transfected with the coding sequence of human OCTN2 (OCTN2-HEK293) were maintained in DMEM supplemented with 10% FBS, 100 units/ml penicillin, 100 μg/ml streptomycin, with Geneticin G-418 at concentration of 600 μg/ml. For cholesterol depletion, cells were exposed for 48 hours to atorvastatin in serum free DMEM medium.

Cytotoxicity assay

OCTN2-HEK293 cells were seeded at the density of 10000 cells/well onto 96-well plates. After 24 hours, cells were treated for 72 hours with increasing extracellular concentrations of atorvastatin. Cell viability was monitored by assessing the cellular redox capacity by alamarBlue® (Thermo Fisher Scientific, Carlsbad, CA).

Uptake assay in intact cells

OCTN2-HEK293 cells were seeded onto 3.5-cm dishes coated with 0.1 mg/ml poly-D-lysine at a density of 3 x 10^5 cells/well. After 72h, cells were exposed for 48 hours to atorvastatin in serum free DMEM medium, washed twice with pre-warmed Na^+-containing transport buffer. Cells were exposed to Na^+-containing transport buffer spiked with [^{3}H]L-carnitine. Uptake was stopped by quick aspiration and extensive washing with ice-cold Na^+-containing transport buffer. Cells were solubilized with 1 ml of 1% (w/v) Triton X-100 solution. A 500 μl-aliquot was assessed for intracellular radioactivity and a 25 μl-aliquot was used for protein determination by the bicinchoninic acid protein assay (Interchim, Montluçon Cedex, France). For determination of OCTN2-independent uptake of L-carnitine, the uptake was measured in Na^+-free transport buffer, in which the Na^+ was replaced with the mono-cation choline, and from the uptake in Na^+-containing transport buffer to determine the L-carnitine OCTN2-mediated transport.

Lipid extraction

Cells were seeded at a density of 3 x 10^5 cells/well onto 6-well plate pre-coated with 0.1 mg/ml poly-D-lysine. After 72 hours, cells were harvested and resuspended in 1 ml of PBS in glass tubes. One hundred μl were lysed with 400 μl of 1% (w/v) Triton X-100 for BCA protein determination. The remaining cell suspension was mixed with 3 ml of chloroform: methanol (2:1) solution spiked with [^{14}C]cholesterol, serving as internal standard.

After 20 minutes in shaking, samples were centrifuged for 5 minutes at 1500g_{av} for phase separation. Upper phase and interphase were discarded and the lower phase containing the lipid fraction was dried under a nitrogen flux at 30°C. Finally, the lipid pellet was resuspended in 300 µl of ice-cold chloroform. A 50 µl-aliquot was used for assessing radioactivity by liquid scintillation counting.

Thin layer chromatography

For the analysis of the cholesterol content, aliquots from the extracted lipids were loaded on HPTLC Silica gel 60 plates with a concentrating zone (Merck KGaA, Darmstadt, Germany) using an automated Camag TLC sampler ATS4 and separated by one-dimensional thin layer chromatography (TLC). Cholesterol was resolved in 62.4% n-hexane, 18.3% n-heptane, 18.3% diethyl ether and 1% acetic acid. Staining was performed in 9.6% orthophosphoric acid (v/v) and 3% copper acetate (w/v), and then the plate was dried at 120-130°C for 30 minutes. Bands were scanned at 366 nm and absolute quantification performed from a serial dilution of cholesterol resolved in parallel. The values were then normalized for the respective [^{14}C]cholesterol levels and the protein content.

Statistical Analysis

Statistical comparisons were performed using GraphPad Prism (version 8.0 for Windows, GraphPad Software). Comparisons between two groups were performed with the two-tailed Student's unpaired t-test.

Figure S1

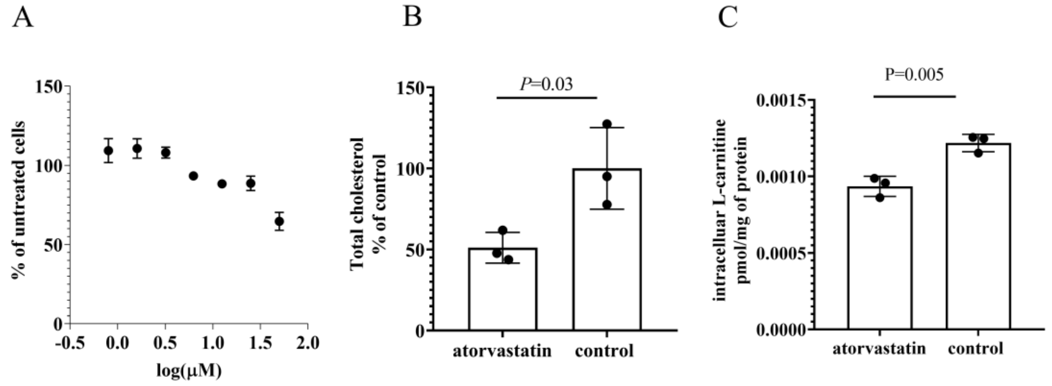

Figure S1. Cell viability, cholesterol content and L-carnitine influx in cells overexpressing the human OCTN2 exposed to atorvastatin. Viability of OCTN2-HEK293 cells incubated for 48h to increasing extracellular concentrations of atorvastatin. Data are expressed as percentage of the untreated control and represent the mean ± S.D. from three independent experiments (A). Total cholesterol level of OCTN2-HEK293 cells exposed for 48h to atorvastatin at the extracellular concentration of 2.5 μM. Data are expressed as percentage of the untreated control and represent the mean ± S.D. from three independent experiments. The indicated P-value was calculated from unpaired t-test comparisons (B). Fifteen-second uptake of L-carnitine at the extracellular concentration of 0.5 μM, in OCTN2-HEK293 cells after 48 hour-incubation with atorvastatin at the extracellular concentration of 2.5 μM. Uptake data were subtracted of the uptake values in Na^+-free buffer and expressed as the mean ± S.D. from three independent experiments. The indicated P-value was calculated from unpaired t-test comparisons (C).
